# Hereditary Spastic Paraplegia Is a Common Phenotypic Finding in ARG1 Deficiency, P5CS Deficiency and HHH Syndrome: Three Inborn Errors of Metabolism Caused by Alteration of an Interconnected Pathway of Glutamate and Urea Cycle Metabolism

**DOI:** 10.3389/fneur.2019.00131

**Published:** 2019-02-22

**Authors:** Emanuele Panza, Diego Martinelli, Pamela Magini, Carlo Dionisi Vici, Marco Seri

**Affiliations:** ^1^Medical Genetics Unit, S. Orsola-Malpighi Hospital, Department of Medical and Surgical Sciences, University of Bologna, Bologna, Italy; ^2^Division of Metabolism, Bambino Gesù Children's Research Hospital, Rome, Italy; ^3^Medical Genetics Unit, Policlinico S. Orsola-Malpighi, Bologna, Italy

**Keywords:** Hereditary Spastic Paraplegia, SPG9, ALDH18A1, P5CS deficiency, arginase deficiency, HHH syndrome

## Abstract

Hereditary Spastic Paraplegias (HSPs) are a clinically and genetically heterogeneous group of neurodegenerative disorders characterized by a progressive rigidity and weakness of the lower limbs, caused by pyramidal tract lesions. As of today, 80 different forms of HSP have been mapped, 64 genes have been cloned, and new forms are constantly being described. HSPs represent an intensively studied field, and the functional understanding of the biochemical and molecular pathogenetic pathways are starting to be elucidated. Recently, dominant and recessive mutations in the *ALDH18A1* gene resulting in the deficiency of the encoded enzyme (delta-1-pyrroline-5-carboxylate synthase, P5CS) have been pathogenetically linked to HSP. P5CS is a critical enzyme in the conversion of glutamate to pyrroline-5-carboxylate, an intermediate that enters in the proline biosynthesis and that is connected with the urea cycle. Interestingly, two urea cycle disorders, Argininemia and Hyperornithinemia-Hyperammonemia-Homocitrullinuria syndrome, are clinically characterized by highly penetrant spastic paraplegia. These three diseases represent a peculiar group of HSPs caused by Inborn Errors of Metabolism. Here we comment on these forms, on the common features among them and on the hypotheses for possible shared pathogenetic mechanisms causing the HSP phenotype.

## Introduction

Hereditary Spastic Paraplegias (HSPs) represent a heterogeneous group of neurodegenerative conditions characterized by a progressive inability to walk due to length-dependent axonal degeneration of the pyramidal tract (1). A simple clinical criteria to classify HSPs is based on the presence of spastic paraplegia as the only clinical sign (“Pure” forms) or the co-presence of additional symptoms (“Complicated” forms) (2).

Despite the fact that more than 80 forms have been mapped, many patients remain without a genetic diagnosis, suggesting that more genes or undefined causes are involved with HSP (3).

Among the Inborn Errors of Metabolism (IEM), spasticity represents a common finding in many forms with pyramidal tract involvement (4). Indeed, the neurons that form these tracts have extremely long axons, and they can be selectively vulnerable to metabolic deregulation resulting in neurodegenerative diseases.

**Figure 1:**
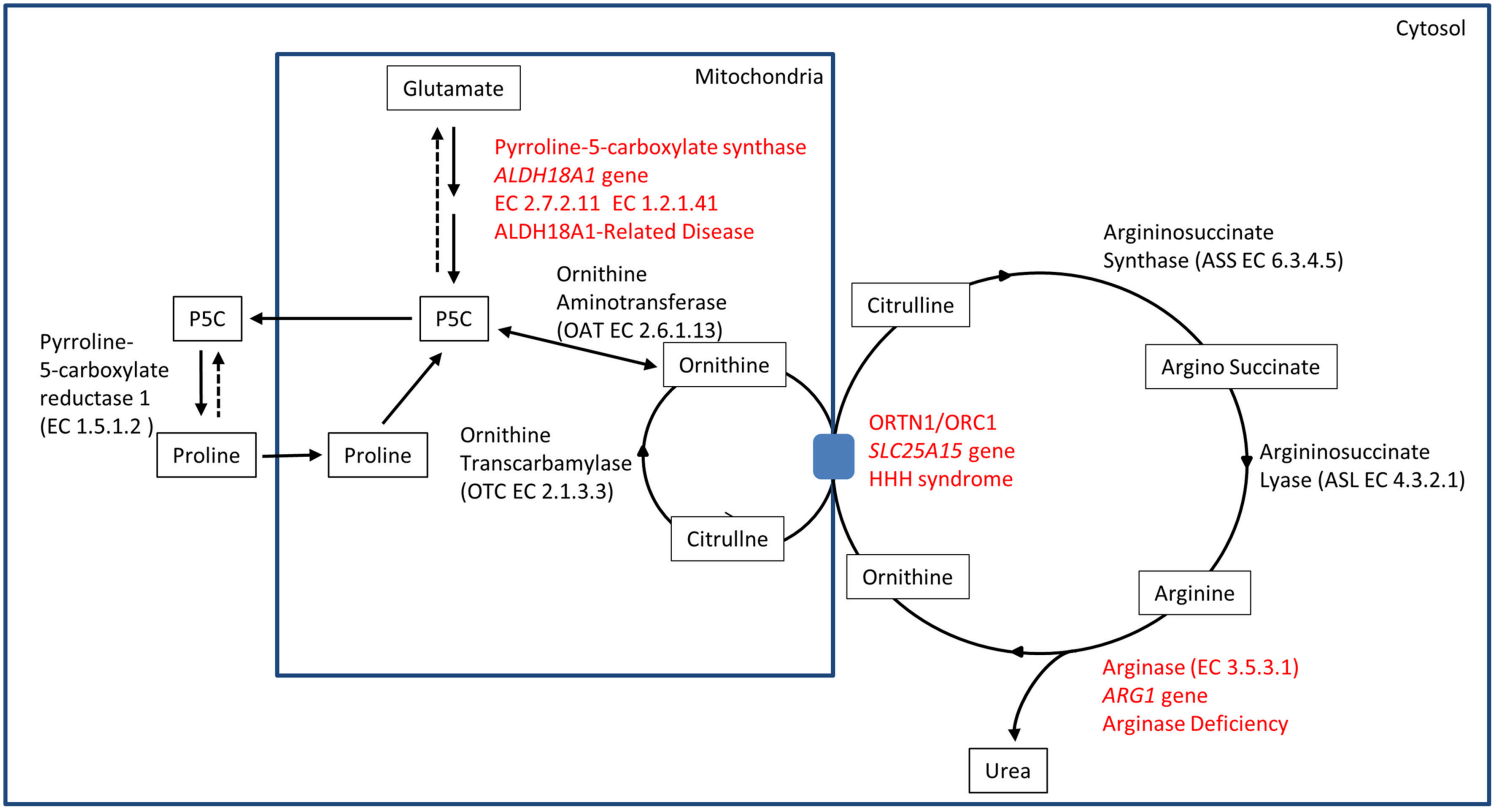
Biochemical pathway.

The identification of *ALDH18A1* as new HSP-disease gene (SPG9) (5, 6) pointed toward the identification of a common biochemical pathway where two other well-known IEM-disease-genes (*SLC25A15* in HHH syndrome and *ARG1* in Argininemia) cause syndromes where spastic paraplegia is present and highly penetrant. This metabolic pathway involves the metabolism of glutamate connected to the urea cycle, thus identifying a subgroup of HSP caused by IEM affecting a common metabolic pathway (Figure 1).

From a clinical point of view, it is important to promptly identify these conditions since for some IEM therapeutic options are available. Furthermore, from a scientific perspective, studying this group of diseases may give a deeper understanding of possible converging mechanisms resulting in the spastic paraplegia phenotype, allowing to design a tailored therapy.

## Phenotypes

### Delta-1-pyrroline-5-carboxylate-synthetase (P5CS) Deficiency

The *ALDH18A1* gene maps on chromosome 10 and it encodes for delta-1-pyrroline-5-carboxylate synthetase (P5CS), an enzyme that localizes in the mitochondria inner membrane.

Mutations in this gene cause P5CS deficiency, a condition first described in 1998 and molecularly characterized in 2000 (7–9).

P5CS is a bifunctional ATP and NADPH dependent enzyme, converting glutamate into L-glutamate-5-semi-aldehyde (GSA) in two steps, catalyzed sequentially by the L-glutamate 5-kinase domain (G5K) (EC2.7.2.11) and by the L-glutamyl-5-phosphate reductase domain (G5PR) (EC1.2.1.41). GSA is in tautomeric equilibrium with delta-1-pyrroline-5-carboxylate (P5C) and is then converted into proline by pyrroline-5-carboxylate reductase or it is directed toward the urea cycle where it is involved in the biosynthesis of ornithine, arginine and citrulline (Figure 1).

Two transcriptional variants of this gene have been described, differing only by two amino acids on protein level. The long form is expressed in several tissues, while the short form is highly expressed in the intestine and it is inhibited by ornithine (10).

Despite the variable clinical presentations among patients with *ALDH18A1* mutations (“*ALDH18A1*-Related Disease”), at least two distinct conditions exist.

## *ALDH18A1* Related Hereditary Spastic Paraplegia

Mutations in *ALDH18A1* can cause dominant (SPG9A, MIM#601162) and recessive (SPG9B, MIM#616586) forms of HSP. These forms are mainly characterized by spasticity of the lower limbs, and the clinical picture can be complicated by low plasma levels of proline, arginine, citrulline, and ornithine associated with hyperammonemia, developmental delay, persistent vomiting, hypotonia, early cataracts and connective tissues abnormalities (5, 6, 11).

## *ALDH18A1* Related Cutis Laxa

Mutations in *ALDH18A1* can cause forms of cutis laxa, inherited as autosomal dominant (AD3, MIM#616603) or autosomal recessive (ARIIIA, MIM#219150) disease. Clinical features may include early cataracts, connective tissues abnormalities, progeroid features, vessels tortuosity, and neuropathy. So far, only two residues have been found mutated in the dominant form (12–14) while, for the recessive form, mutations have been identified in different parts of the protein.

A clear genotype-phenotype correlation for the distinct *ALDH18A1*conditions is not yet apparent.

## Hyperornithinemia-Hyperammonemia-Homocitrullinuria Syndrome (HHH Syndrome)

The association of hyperornithinaemia, hyperammonaemia, and homocitrullinuria is pathognomonic for HHH syndrome (MIM#238970), an autosomal recessive disease caused by biallelic mutations in *SLC25A15* gene (alias *ORNT1*, MIM#603861). This gene maps on 13q14.11, and it encodes for the mitochondrial ornithine/citrulline antiporter ORC1. Mutations in this gene result in a defect of ornithine transport through the mitochondrial membrane (Figure 1), causing a functional deficiency of the urea cycle. This mechanism results in the increase of ornithine levels in cytosol (and in plasma), while causing ornithine deficiency inside mitochondria, affecting the urea cycle. The latter situation leads to the accumulation of carbamoylphosphate, which is shifted to the formation of orotic acid by an alternative pathway, and induces the formation of homocitrulline from lysine by ornithine transcarbamylase.

HHH can occur at any age (15–17). The clinical presentation of HHH syndrome covers a broad spectrum of symptoms, including protein intolerance, vomiting, seizures, confusion, and developmental delay. The most severe forms have been reported with neonatal onset of lethargy, hypotonia, and seizures developing into coma and even death (18). There are also slowly/chronic progressive forms, characterized by the patients aversion to food rich in proteins, variable intellectual disabilities and/or cognitive regression, and signs of motor deficit (18).

Most patients develop neurological dysfunction mainly characterized by pyramidal tract signs with spastic gait, associated with cerebellar symptoms (Table 1).

**Table 1:** Main features found in P5CS deficiency, ARG1 Deficiency, and HHH syndrome.

	**P5CS deficiency**		**HHH**	**ARG1 deficiency**
	**HSP**	**CL**		
**Inheritance**	**AR/AD/*****de novo***		**AR**	**AR**
**MAJOR CLINICAL FEATURES**				
Intellectual disability	+	+	+	+
Retina degeneration	+	–	±*	–
Cataract	+	+	–	–
Persistent vomiting	+	±	+	+
Developmental delay/MR	+	+	+	+
Seizures	±	–	+	+
Cerebellar ataxia	±	–	+	–
Thin corpus callosum	±	–	–	–
Pyramidal signs/spastic paraparesis	+	±	+	+
Dysmorphisms	±	+	–	–
Progeroid appearance	–	+	–	–
Microcephaly	±	-	–	–
Lax and wrinkled skin	–	+	–	–
Visible vein	–	+	–	–
Joint laxity	+	+	–	–
Liver dysfunction	–	–	+	–
Episodic lethargic coma	–	–	+	+
**BIOCHEMICAL ALTERATIONS**				
Plasma ammonia	↑± (fasting)	–	↑+ (fed)	↑+ (fed)
Plasma ornithine	↓±	↓±	↑+	–
Plasma arginine	↓±	↓±	–	↑+
Plasma citrulline	↓±	↓±	↓±	–
Plasma proline	↓±	–	–	–
Homocitrullinuria	–	–	+	–
Orotic aciduria	–	–	+	+

*HSP, Hereditary Spastic Paraplegia; CL, Cutis Laxa*.

* *one report only*.

## Arginase Deficiency (ARG1 Deficiency)

Arginase deficiency (MIM#207800) is a recessive condition caused by mutations in *ARG1*, a gene mapping on chromosome 6q23.2. This gene encodes for arginase, the last enzyme of the urea cycle, which is necessary for the transformation of arginine into urea and ornithine (EC3.5.3.1) (Figure 1). Arginase protein is a homotrimer in physiological state, and several detrimental mutations have been identified. Arginase deficiency results in hyperargininemia, with elevated levels of arginine in plasma and other fluids. The accumulation of arginine leads to the use of alternative pathways for arginine metabolism. Some of these alternative pathways are not fully understood. Accumulation of metabolites as guanidine compounds, nitric oxide and homoarginine concentration have been observed. As a consequence, these metabolites could have a pathological role (19).

Usually urea cycle disorders present symptoms at birth, but in case of arginase deficiency, first symptoms are often noted between 2 and 4 years of age and consist of a variable association of progressive spastic paraplegia, intellectual disability and seizures (20, 21). Short stature and failure to thrive may also be present. In some cases, paraparesis may appear in adolescents or young adults (20, 22). Only a minority of patients show signs of protein intolerance, and ammonia is often normal or mildly increased.

## Animal Models

Engineered organisms are indispensable tools to model genetic conditions in order to dissect their pathological mechanism and to test innovative therapies at the entire organism level (23).

## Model Organisms for HHH and P5CS Deficiency

As for HHH syndrome, the disease does not seem to be only an exclusive prerogative of the human species, since a spontaneous animal model exists. A report describes two consanguineous weanling foals, presenting a subacute encephalopathy in the early post-weaning period (24). The clinical and biochemical picture strikingly resembled those of human HHH syndrome, with anorexia, poor growth, abnormal behavior, bilateral forelimb, and hindlimb ataxia, and circling. The biochemical profile was characterized by hyperammonemia, liver dysfunction, reduced blood urea nitrogen, elevated levels of ornithine and glutamine in serum and increased orotic acid excretion in urine.

To this day, engineered animal models for HHH syndrome have not been reported, and models for *ALDH18A1*-related disease have not yet been described. The research in the field would greatly benefit from the generation and characterization of such models.

## *Arg1* Deficiency Model Organisms

Two isoforms of arginase exist: *ARG1* is the cytoplasmic form, mainly expressed in the liver. *ARG2* is expressed in the mitochondria and it is expressed mainly in non-hepatic tissues.

*Arg1* and *Arg2* are the mouse orthologs of the human *ARG1* and *ARG2* genes.

*Arg1*-KnockOut (KO), and double *Arg1* and *Arg2*-KO mice have been described (25–27). One *Arg1*-KO mouse has been generated by inserting a Neomycin resistant gene in place of exon 4 of the endogenous *Arg1* gene. The resulting homozygous KO animals completely lacked liver arginase activity, exhibited severe symptoms of hyperammonemia and died between post-natal day 10 and 14, thus sharing several features of the human condition (25). Another KO mouse model (27) showed an accumulation of several guanidino compounds, as direct or indirect metabolites of arginine metabolism. The very same compounds are elevated in the blood of uremic patients and in the plasma and cerebrospinal fluid of hyperargininemic patients, suggesting that these compounds could represent the neuropathogenetic agents responsible for neurological complications in Arginase deficiency. Indeed, the guanidino compounds alpha-keto-delta-guanidinovaleric acid, alpha-N-acetylarginine, and argininic acid were increased in brain tissue from the *Arg1*-deficient mouse model of hyperargininemia. Several guanidino compounds were also elevated in plasma, liver, and kidney (27).

Double KO for *Arg1* and *Arg2* presented with the same phenotype of *Arg1* KO, but showed increased plasma level of arginine and decreased plasma levels of ornithine. Ornithine and arginine were altered also in other tissues, indicating that the deficiency of ornithine has a causative role for the fatal hyperammonemia in the mice (26).

## Therapy

Drugs are necessary to treat promptly and effectively the sensitive organs affected in these conditions. The biochemical characteristics of this group of disorders make them good candidates to test therapies based on aminoacids supplementation. Alternative therapies should also be evaluated and tested. For these reasons, the development of *in vitro* and especially *in vivo* models is essential.

As for P5CS deficiency, Baumgartner reported on the use of ornithine supplementation, attempted in a patient of 12 years of age, who was presenting with progressive neurological deterioration. This approach, in the specific context of the reported article, did not significantly modify the progression of symptoms of the patient (9).

Another patient was treated with arginine supplementation, and this approach was attempted because brain creatine was decreased, as detected by proton magnetic resonance spectroscopy (H-MRS) (28). The endogenous synthesis of creatine is critical for the brain, and a decrease of its rate-limiting precursor may lead to a suboptimal creatine synthesis. This therapeutic approach improved metabolic parameters and an amelioration of the psychomotor symptoms was noted over the time of the study (28).

Similarly to other urea cycle defects (29), treatment in HHH syndrome and Arginase deficiency is based on a low-protein diet combined with the use of ammonia scavengers sodium benzoate, sodium phenylbutyrate or glycerol triphenylbutyrate. In HHH syndrome, treatment relies also on the use of citrulline, arginine, or ornithine supplementation (18).

Pharmacological and dietary treatments are the standard clinical approach for these disease and reduce the risk of metabolic decompensation. The progression of spastic paraplegia, however, is unaffected (18, 21).

## Pathogenetic Mechanisms

The pathogenesis of neurological manifestations in patients with P5CS deficiency, Arginase deficiency and HHH syndrome, is not completely understood and may be related to different mechanisms.

### Arginine Imbalance

Arginase deficiency is characterized by very high plasma levels of arginine and decreased ornithine recycling. Both these biochemical features can be responsible for the phenotype. It has been suggested that increased levels of arginine can be responsible for spasticity and other severe cerebral and motor neurological signs. Arginine and its metabolites, including guanidino compounds, are reported to act as neurotoxins (21). Guanidino compounds can indeed cause demyelination with consequent upper motor neuron signs, and they can be responsible for the epileptic crisis (21). In addition, arginine is the substrate for nitric oxide synthase, generating oxidative damage that can affect neuronal survival (30). Patients diagnosed and treated since birth with protein restriction and essential aminoacid supplementation rarely present with metabolic decompensation, but they do not have completely normalized arginine levels, despite normal ammonia in blood (18, 21). This suggests that chronically elevated levels of arginine may play a direct role in the neuropathologic manifestations.

### Ammonia Toxicity and Deregulation of Proline Biosynthesis

In HHH syndrome, hyperammonemia occurs due to the inability to import ornithine from the cytosol into the mitochondria resulting in a functional impairment of the urea cycle at the level of ornithine transcarbamoylase. In the absence of intramitochondrial ornithine, accumulating carbamyl phosphate either condenses with lysine to form homocitrulline, leading to homocitrullinuria, or is shunted through the cytosolic pyrimidine biosynthetic pathway leading to increased excretion of orotic acid and uracil in the urine (31).

In HHH syndrome, abnormal mitochondria are often seen, suggesting a role of a functional defect at mitochondrial level.

Hyperammonemia may be involved in CNS pathogenesis, since it causes neurodegeneration due to increased production of reactive oxygen species and decreased activities of free radical scavenging enzymes, representing a link between common CNS disorders and some IEM. Nevertheless, as in the case of Argininemia, it is unlikely that hyperammonemia *per-se* is solely responsible for the pathophysiology of this disorder, since also affected individuals who are diagnosed early and maintain good metabolic control and normal plasma ammonia levels develop progressive neurological dysfunction years after the initial diagnosis (32).

Therefore, other metabolic factors including persistent or acute accumulation of ornithine and homocitrullinemia may possibly contribute to the neurological symptoms, typical of patients affected by this disorder (33).

Indeed, the first patient reported with recessive mutations in *ALDH18A1* causing P5CS deficiency had a clear biochemical phenotype with low levels of plasma ornithine, citrulline, arginine, proline, and fasting hyperammonemia possibly reflecting the need of *de novo*-synthetized ornithine for ureagenesis under fasting conditions (8, 9, 28).

These manifestations were largely interpreted as being connected to the deficient biosynthesis of ornithine/arginine and proline (8). In fact, some aspects of the phenotypic manifestation can be explained by deficient proline synthesis and in a loss of proline rich proteins, such as collagen.

Interestingly, PYCR1 deficiency (PYCR1D) presents some similarities with P5CSD (34). PYCR1 (MIM#179035) is an enzymes that catalyzes the final step of proline biosynthesis and reduces pyrroline-5-carboxylate (P5C) to L-proline (EC 1.5.1.2). PYCR1D causes autosomal recessive forms of cutis laxa (ARCL2B and ARCL3B, MIM#612940 and MIM#614438, respectively). Common features of P5CSD and PYCR1D include connective tissues defects (loose inelastic skin, joint laxity, progeroid features) and developmental delay.

Despite the enzymatic deficiency, PYCR1D patients do not present plasma aminoacid abnormalities. In particular, plasma proline levels are normal or slightly toward the lower limit. The absence of such alterations could be the result of a compensative effect due to the presence in humans of PYCR2 and PYCRL paralogous genes (34). P5CS and PYCR1 enzymes localize in the inner membrane of mitochondria. In the case of PYCR1D, patients show mitochondrial abnormalities as demonstrated by experiments in patient's fibroblasts cultured under oxidative stress conditions (28). However, similar mitochondrial alterations have not been identified in patients with P5CS deficiency, supporting a distinct pathogenesis in these two diseases and pointing toward a block of ornithine/arginine and proline metabolism as the main mechanism in P5CS deficiency (34).

### Abnormal Creatine Synthesis

A specific decrease in brain creatine peak has been shown in P5CSD patients (28), by H-RMS. This is an interesting observation, given the importance of arginine for creatine synthesis (35) and the association of brain creatine deficiency with developmental delay, hypotonia, mental retardation, poor speech development, seizures, and brain atrophy (28, 36). Possibly, the decrease in brain creatine may reflect the presence of suboptimal arginine levels in the brain, and this could have disastrous effects given the importance of endogenous creatine synthesis in this organ (28). Secondary creatine deficiency has also been observed in HHH (15, 37) due to low cellular arginine availability and possibly inhibition of creatine biosynthesis because of ornithine excess (37). In Argininemia, markedly elevated arginine levels may result in higher concentrations of guanidinoacetate and higher rates of creatine synthesis (38).

### Effects on Autophagy

Interestingly, autophagy has been recently linked to ammonia detoxification (39). Autophagy, moreover, is crucial for the development of central nervous system and for neuronal function, and some HSPs are due to genetic defects linked to autophagy machinery (40). Mutations in one of the four subunits of the adaptor protein complex 4 (AP4), a heterotetrameric protein that regulates the transport of membrane proteins, lead to rare forms of HSP (SPG47, SPG50, SPG51, SPG52) (41). All these disorders share numerous similarities; therefore, they are collectively designed as “AP-4 deficiency syndrome,” which belong to the group of the “Adaptinopathies” (41). AP-4 complex is involved in transport between the Trans-Golgi network and endosomes, contributing to polarized sorting in neurons and the development/integrity of neural network (41). In particular, AP4 complex promotes signal-mediated export from the trans-Golgi network to the peripheral cytoplasm of ATG9A, a protein critical for the maturation of preautophagosomal structures (42). Similarities with AP4-related HSP suggest a potential contribution of autophagy also to the pathogenesis of neuronal damage in HHH syndrome, Argininemia and P5CS deficiency, linking their phenotypic manifestations. A common mechanism could be represented by arginine level imbalance (usually low in P5CSD and HHH syndrome, high in Argininemia).Under this respect, post-translational arginylation of proteins is an important regulator of many physiological pathways in cells, both in basal condition and in neurodegenerative processes. Arginylation is involved in signaling processes of proteins and polypeptides that are further ubiquitinated and degraded by the proteasome and is implicated in autophagy/lysosomal degradation pathway (43). Brain arginine metabolism is dramatically altered in Alzheimer disease (44). Arginine imbalance, moreover, is a known modulator of autophagy in cancer cells, and depletion of ASS1 (argininosuccinate synthetase), the main enzyme involved in arginine synthesis, led to inhibition of tumor growth and decreased cell invasion via induction of autophagy-lysosome machinery (45). It has also been demonstrated *in vitro* that high arginine down-regulates ASS1 expression (46).

### New Perspectives

The endoplasmic reticulum (ER) is the biggest organelle in cells and formation and maintenance of ER morphology are regulated by a series of proteins controlling membrane fusion and curvature. Some of these regulators have been demonstrated to be involved in HSPs, in particular Reticulons (RTNs) family. RTNs are a group of membrane associated proteins involved in shaping the tubular endoplasmic reticulum network, membrane trafficking, inhibition of axonal growth, and apoptosis. (47, 48). Considering the relevance of metabolic signals in modulating endoplasmic reticulum responses in normal and stress conditions, we can expect for the future a crescent evidence of a role of this class of proteins in the pathogenesis of inborn errors of metabolism associated with HSPs and axon degeneration.

## Concluding Remarks

The development of a neurological phenotype in HHH syndrome, ARG1 and P5CS deficiency, can be induced by the formation of toxic compounds, resulting from the accumulation of substrates, or alteration in mitochondria, where ornithine is low or absent in these conditions. These observations point toward an impairment of the ornithine/arginine metabolism as a common mechanism for the development of the neurodegenerative phenotype observed in all three metabolic HSPs.

Moreover, a link between autophagy and HSP has been demonstrated (40). Strong evidence support a role of arginine deregulation and autophagy in cancer and it appears to be involved also in the pathogenesis of neurodegenerative disorders. Thus, it is possible that alteration of arginine levels, common in the three conditions, can deregulate autophagy.

To test this hypothesis it would be ideal to perform a standardized analysis of these patients including an accurate clinical evaluation (especially necessary for P5CS deficiency, where the clinical heterogeneity is high), plasma aminoacid profile analysis in fasting and non-fasting conditions, and H-RMS to check for alterations in *in vivo* metabolite concentrations in the central nervous system.

In order to dissect the pathogenetic mechanism of these conditions, it will also be essential to generate organisms and cell models.

In particular, testing and comparing the metabolomics profile in normal and stress conditions of either patient's primary cell lines or engineered cell lines obtained taking advantage of genome editing technologies will be relevant to understand deregulated pathways. Through a metabolomic approach, it will be possible to demonstrate common abnormalities, pinpointing to a possible therapeutic target.

Finally, the generation of model organisms will be essential in order to test and evaluate the efficacy of the therapeutic approach *in vivo*.

## Author Contributions

EP, DM, CD, and MS conceived the study. EP, PM, and DM collected and interpreted the literature data. EP, DM, CD, and MS wrote the manuscript.

### Conflict of Interest Statement

The authors declare that the research was conducted in the absence of any commercial or financial relationships that could be construed as a potential conflict of interest.
